# Engaging youth as leaders and partners can improve substance use prevention: a call to action to support youth engagement practice and research

**DOI:** 10.1186/s13011-023-00582-7

**Published:** 2023-11-27

**Authors:** Parissa J. Ballard, Heather K. Kennedy, Jessica J. Collura, Elena Vidrascu, Chelsey Garcia Torres

**Affiliations:** 1 Family & Community Medicine, Wake Forest University School of Medicine, 1920 West 1st St, Piedmont Plaza, Building 1, Winston-Salem, NC 27104 USA; 2 Community & Behavioral Health, Colorado School of Public Health, University of Colorado, Denver, USA; 3 Ohio Education Research Center, John Glenn College of Public Affairs, The Ohio State University, Columbus, USA; 4 Psychology & Neuroscience Department, University of North Carolina at Chapel-Hill, Chapel-Hill, USA; 5 Center for Public Health Practice, Colorado School of Public Health, Bachelor of Science Psychology, Denver, USA

**Keywords:** Youth engagement, Substance use prevention, Adolescents, Workforce development

## Abstract

**Background:**

As a subfield of prevention science, substance use prevention researchers and professionals are increasingly focused on translating research into practice, developing the workforce of prevention specialists, and creating a robust prevention infrastructure. One critical need for professional development among the substance use prevention workforce is training and technical assistance around how to include young people in developing, implementing, and evaluating substance use prevention programs.

**Main body:**

Amplifying youth voices can increase the quality and responsiveness of youth prevention research and practice, as well as hasten and improve the translation of prevention interventions into practice while also benefiting youth themselves. Yet, youth engagement is multi-layered and nuanced. Training prevention professionals who work with youth in *youth development* and *youth/adult partnerships* is critical to support meaningful youth engagement efforts. We assert that the substance use prevention workforce needs at least three specific competencies to engage youth meaningfully in prevention: 1) understand adolescent development and the core elements of youth-adult partnerships; 2) apply this knowledge to program design and practice; and 3) implement relational practices to share power with young people.

**Conclusion:**

Incorporating the insights of young people can improve substance use prevention. The substance use prevention workforce should be supported in developing competencies to meaningfully engage youth. These competencies require training, and resources must be devoted to support appropriate training.

## Background

The field of prevention science is instrumental in designing, implementing, evaluating, and disseminating evidence-based programs and interventions to prevent substance use among young people, to reduce harms associated with substance use, and to promote health [1]. Thought leaders in substance use prevention science argue that the field should focus on developing the workforce of prevention specialists [1] and a robust prevention infrastructure [2] in order to translate prevention science into services [3]. This brief comment is meant for researchers and practice-oriented professionals focused on improving substance use prevention practice; we offer specific competencies that are needed for the prevention workforce in order to meaningfully engage youth in prevention. We assert that training and funding should be provided for the prevention workforce to develop these competencies.

## Main text

### Youth engagement definition and evidence for multi-level benefits

Within the field of substance use prevention, Youth Engagement (YE) can be thought of as an approach in which prevention organizations effectively engage youth as leaders or partners in planning, tailoring, implementing, and/or evaluating prevention programming [4]. There is a strong theoretical justification [5], real-world precedent [6], and evidence supporting [7–10] the practice of engaging youth as active co-constructors in community initiatives where youth are a central constituency. We define youth broadly as those between the ages of 11—25, sometimes referred to as adolescents and young/emerging adults [11, 12] and we use the term “substance use” to refer to a broad array of substances that practitioners aim to prevent and to reduce associated harms for young people. Core to youth engagement is youth-adult partnerships, which operationalizes partnerships as work that is 1) collective, 2) impactful, and 3) distributed based on skills, expertise, networks, and interests of all involved parties [13]. Centering youth as co-constructors shifts the perspective away from youth as passive recipients of policies or programs.

Calls for increased youth participation in public health emanate from rights-based perspectives arguing for participation as a human right, as well as from empirical perspectives arguing that participation improves outcomes [12, 14]. Studies suggest that youth engagement can increase the quality, responsiveness, and reach of community initiatives [9]. Many programs and organizations that partner with youth showcase examples of how youth involvement increases the quality and relevance of prevention programming [9, 10, 12]. Further, peer-led substance use prevention interventions have been found to reduce tobacco, alcohol, and cannabis use [11] and raise community awareness of substance use and related solutions [9]. Importantly, youth benefit by taking active roles in the spectrum of health prevention and promotion activities. Specifically, they can experience empowerment, contribute to their communities, form positive connections with adults while gaining personal skills (e.g., communication and leadership) and professional benefits (e.g., broadened networks) [9, 15]. Engaging the constituents affected by research and programming has a long history under the umbrella of participatory approaches (e.g., community-based participatory research, community coalitions) and increasingly is recognized as a practice with the potential to support equity and disrupt traditional power dynamics, including between youth and adults.

Engaging youth in a meaningful way in public health, especially in the United States, is challenging as these efforts must have the adult and organizational capacity and readiness to share power with youth, and to address logistical, developmental, and systemic barriers that young people face [7, 16, 17]. Furthermore, in the United States, adults are afforded power over young people’s lives, as the United States has yet to ratify the Convention on the Rights of a Child [18]. Adults need awareness of their own attitudes and beliefs about young people’s capacity as well as the skills to lead while amplifying young people’s needs within and outside of the partnership [19]. Thus, engaging youth meaningfully requires a proactive and thoughtful approach accompanied by the resources, training, and support necessary to effectively improve prevention and empower young people.

### Youth engagement in substance use prevention practice

YE in substance use prevention practice is critical for several reasons. Young people offer essential perspectives as they are embedded in youth culture and have first-hand knowledge of the attitudes and behaviors of their peers. YE can improve prevention initiatives that target multiple different substances simultaneously, as is often the case in prevention practice, or in substance-specific initiatives. Further, because youth characteristics and environmental contexts differentially affect motivations for substance use and attitudes about prevention programs, incorporating local youth perspectives, especially from youth with lived experiences with substance use, when planning and tailoring prevention efforts can increase the responsiveness and uptake of prevention programs. Existing youth engagement structures in substance use prevention practice include coalitions, youth advisory boards, participatory action research, and youth participating as paid consultants or evaluators.

There is broad recognition of the value of youth engagement in substance use prevention research and practice [9, 11], and many practical resources are available to guide practitioners in involving young people in health policy and promotion. For example, the National Network of State Adolescent Health Coordinators (NNSAHC) offers resources about youth engagement, as does the Substance Abuse and Mental Health Services Administration (SAMSHA) and Community Anti-drug Coalitions of America (CADCA, see Table 1). YE has been implemented in many areas of public health, including to some extent in substance use prevention, harm reduction, treatment, and recovery programs [9, 11, 12] for example using peer support models [20]. Toolkits focused on prevention and harm reduction that were developed or co-developed by young people are available [21]. However, YE remains an innovative strategy underutilized by substance use prevention organizations and warrants more empirical examination to document its effects on prevention. Given increasing recognition of YE as a valuable strategy, with high potential to provide multi-level benefit to both youth and prevention efforts, practitioners should be supported to gain the skills and resources for allocating effort towards YE.

**Table 1:** Further Resources for Youth Engagement

Organization	Resource website (links live as of August 2023)
National Network of State Adolescent Health Coordinators (NNSAHC)	https://nnsahc.org/key-topics/youth-engagement/
Substance Abuse and Mental Health Services Administration (SAMSHA)	https://store.samhsa.gov/sites/default/files/SAMHSA_Digital_Download/sma16-4985.pdf
Community Anti-drug Coalitions of America (CADCA) and Office of National Drug Control Policy (ONDCP)	https://www.cadca.org/resource/youth-engagement-series/

Importantly, YE is aligned with existing and evolving competency standards in the field of prevention. SAMHSA’s *Prevention Core Competencies* [22] identifies cultural inclusion, or the “ability to ensure that those affected by a problem are an integral part of devising and implementing the solution” (p.32), as a core competency for the prevention workforce. This includes young people who are often the focal population of substance use prevention initiatives. SAMHSA also identifies “age-appropriate and culturally relevant communications with children and youth” as another core competency for the prevention workforce. At an international level, the United Nations Office on Drugs and Crime’s International Standards on Drug Use Prevention highlight the importance of YE. Specifically, resolution 63/4 focuses on “promoting the involvement of youth in drug prevention efforts” [23]. Prevention specialists, in the United States and internationally, are expected to have the competencies needed to engage young people in prevention efforts; however, little training is offered to support professional’s knowledge, skills, and self-efficacy for partnering with young people.

## Conclusions: call to action

As substance use prevention science increasingly focuses on translating science into practice and supporting workforce development efforts, focusing on youth engagement has the potential to support and empower youth and strengthen prevention quality, reach, and responsiveness. In order to move beyond articulating support for YE to living out that commitment, there is a need to train substance use prevention professionals in *youth development* and *youth/adult partnerships.* Despite general enthusiasm for youth engagement, translating this into day-to-day practice that fits within the competing priorities and the requirements and constraints of the substance use prevention funding landscape is challenging [10]. There is a paucity of training specifically for the nuance and messiness associated with “living out” youth-adult partnerships [24]. Yet, training and ongoing coaching are recognized as important factors in youth program effectiveness [25]. In one study, adults significantly increased their confidence to execute youth-engaged activities in systemic approaches to community social action after participating in a youth engagement training course [26]. In order to increase the capacity of the substance use prevention workforce, we believe that the following three competencies, based in youth worker professionalization and practices [27], are foundational for youth engagement training:

1. Understand adolescent development across key domains (e.g., physical, emotional, social, cognitive, emotional, spiritual, civic, identity) and the core elements of youth-adult partnerships (authentic decision making, natural mentors, reciprocal activity, community connectedness) [13]; and
2. Apply this understanding of adolescent development and youth-adult partnerships to the design of youth/adult meetings, materials, and programs that serve young people; and
3. Implement key relational practices (e.g., support youth to make decisions, engage in reciprocal communication, work jointly, share power) that enable authentic collaboration between youth and adults [7, 13, 28].

Training programs focused on these competencies should encourage prevention professionals to reflect on their identities and positionality (i.e., the social identities related to young people they work with) including potential personal biases they may hold toward young people who use drugs. These aspects of identity inform how adults work with young people and self-reflection can minimize potential to harm young people and their communities that can result from biases. Some training and professional support programs that draw on empowerment-based models for engaging young people, such as the Social Justice Approach to Youth Engagement online course [8] and the Dover Youth 2 Youth [29] program, have high potential to improve substance use prevention through building prevention workforce capacity. Specifically, such programs offer the opportunity to develop skills for authentically partnering with young people to co-create solutions to issues identified by youth or through youth-adult partnerships. Given that there are scant opportunities for professional development for authentic youth engagement, more resources are needed to develop, implement, and evaluate training designed around these competencies. Growing the capacity of the prevention workforce for youth engagement will benefit the workforce, their prevention efforts, and ultimately the young people they serve.

## Data Availability

Not applicable.

## Abbreviations

CADCA: Community Anti-drug Coalitions of America
NNSAHC: National Network of State Adolescent Health Coordinators
SAMHSA: Substance Abuse and Mental Health Services Administration
YE: Youth Engagement

## References

[1] Sloboda Z, Johnson KA, Fishbein DH, Brown CH, Coatsworth JD, Fixsen DL, Kandel D, Paschall MJ, Silva FS, Sumnall H, Vanyukov M (2023). Normalization of prevention principles and practices to reduce substance use disorders through an integrated dissemination and implementation framework. Prev Sci.

[2] Volkow N. Creating Sustainable Homes for Prevention Services. National institute of drug abuse. 2023. Available from: https://nida.nih.gov/about-nida/noras-blog/2023/04/creating-sustainable-homes-prevention-services. Cited 2023 Aug 8.

[3] Goldstein AB, Oudekerk BA, Blanco C (2023). From prevention science to services: identifying paths to sustainable evidence-based preventive interventions. Psychiatr Serv.

[4] Ballard PJ, Hernandez GC, Pankratz M, Daniel J, Ross JC, Wagoner K, Moore JB, Rhodes SD (2023). Engaging youth to improve substance misuse prevention: Information guide series.

[5] Ozer EJ, Abraczinskas M, Duarte C, Mathur R, Ballard PJ, Gibbs L, Olivas ET, Bewa MJ, Afifi R (2020). Youth participatory approaches and health equity: Conceptualization and integrative review. Am J Community Psychol.

[6] Collura JJ, Raffle H, Collins AL, Kennedy H (2019). Creating spaces for young people to collaborate to create community change: Ohio’s youth-led initiative. Health Educ Behav.

[7] Dunne T, Bishop L, Avery S, Darcy S (2017). A review of effective youth engagement strategies for mental health and substance use interventions. J Adolesc Health..

[8] Kennedy H, Elnicki J, Torrieri D, Scallan Walter E (2022). Increasing the capacity of youth-serving professionals: Evaluation of the online social justice approach to youth engagement course. Pedagogy Health Promot.

[9] Valdez ES, Skobic I, Valdez L, O Garcia, D, Korchmaros J, Stevens S, ... & Carvajal S. Youth participatory action research for youth substance use prevention: a systematic review. Subst Use Misuse. 2020;55(2), 314–328. 10.1080/10826084.2019.1668014. 10.1080/10826084.2019.1668014 PMC8034545 31596160

[10] Ballard PJ, Hernandez GC, Pankratz MM, Ross JC, Wagoner KG, Moore JB, ... & Rhodes SD.. Engaging youth in substance misuse prevention within state prevention systems: provider perspectives. Health Behav Policy Rev. 2022;9(4):933–948. https://doi.org/10.14485/hbpr.9.4.2. 10.14485/hbpr.9.4.2 PMC10134771 37124425

[11] MacArthur GJ, Harrison S, Caldwell DM, Hickman M, Campbell R (2016). Peer-led interventions to prevent tobacco, alcohol and/or drug use among young people aged 11–21 years: a systematic review and meta-analysis. Addiction.

[12] Aresi G, Ferrari V, Marta E, Simões F (2023). Youth involvement in alcohol and drug prevention: a systematic review. J Community Appl Soc Psychol.

[13] Zeldin S, Christens BD, Powers JL (2013). The psychology and practice of youth-adult partnership: bridging generations of youth development and community change. Am J Community Psychol.

[14] Ozer EJ, Afifi R, Gibbs L, Mathur RT (2018). Youth engagement and participation: Field-building across research and practice. J Adolesc Health.

[15] Anyon Y, Bender K, Kennedy H, Dechants J (2018). A systematic review of youth participatory action research (YPAR) in the United States: Methodologies, youth outcomes, and future directions. Health Educ Behav.

[16] Jacquez F, Vaughn LM, Wagner E (2013). Youth as partners, participants or passive recipients: A review of children and adolescents in community-based participatory research (CBPR). Am J Community Psychol.

[17] Ballonoff Suleiman A, Ballard PJ, Hoyt LT, Ozer EJ (2021). Applying a developmental lens to youth-led participatory action research: A critical examination and integration of existing evidence. Youth Soc.

[18] Assembly UG (1989). Convention on the Rights of the Child. UN Treaty Ser.

[19] Mitra DL (2005). Adults advising youth: Leading while getting out of the way. Educ Adm Q.

[20] Paquette KL, Winn LA, Wilkey CM, Ferreira KN, Donegan LR (2019). A framework for integrating young peers in recovery into adolescent substance use prevention and early intervention. Addict Behav.

[21] Get Sensible. Retrieved 11/14/23 from: https://getsensible.org/about/.

[22] Substance Abuse and Mental Health Services Administration (2021). Prevention Core Competencies.

[23] United Nations Office on Drugs and Crime’s International Standards on Drug Use Prevention. Resolution 63/4: promoting the involvement of youth in drug prevention efforts. 2020. https://www.unodc.org/documents/commissions/CND/Drug_Resolutions/2020-2029/2020/Resolution_63_4.pdf

[24] Kennedy H (2018). How adults change from facilitation youth participatory action research: Process and outcomes. Child Youth Serv Rev.

[25] Durlak JA, DuPre EP (2008). Implementation matters: a review of research on the influence of implementation on program outcomes and the factors affecting implementation. Am J Community Psychol.

[26] Kennedy H, Elnicki J, Walter ES. Increasing the capacity of youth-serving professionals: evaluation of the online social justice approach to youth engagement course. 2021;8(1). 10.1177/23733799211053883.

[27] Vance F (2010). A comparative analysis of competency frameworks for youth workers in the out-of-school time field. Child Youth Care Forum.

[28] Malorni A, Lea CH, Richards-Schuster K, Spencer MS (2022). Facilitating youth participatory action research (YPAR): a scoping review of relational practice in U.S. Youth development & out-of-school time projects. Child Youth Serv Rev..

[29] Mitchell D, Hebert V. One voice youth empowerment model: building knowledge, skills, and advocacy among. https://dovery2y.org/wp-content/uploads/One-Voice-Youth-Empowerment-Model-Long-Term-Evaluation-01262016-wtih-Appendix.pdf. 2016. Accessed 10.24.23.

